# A Sensitive and Robust High-Throughput Screening Assay for Inhibitors of the Chikungunya Virus nsP1 Capping Enzyme

**DOI:** 10.1371/journal.pone.0158923

**Published:** 2016-07-18

**Authors:** Kristen M. Bullard-Feibelman, Benjamin P. Fuller, Brian J. Geiss

**Affiliations:** 1 Department of Microbiology, Immunology, and Pathology, Colorado State University, Fort Collins, Colorado, United States of America; 2 Department of Biochemistry and Molecular Biology, Colorado State University, Fort Collins, Colorado, United States of America; 3 School of Biomedical Engineering, Colorado State University, Fort Collins, Colorado, United States of America; CEA, FRANCE

## Abstract

Chikungunya virus (CHIKV) is a mosquito-borne *Alphavirus* that causes severe and debilitating disease symptoms. Alarmingly, transmission rates of CHIKV have increased dramatically over the last decade resulting in 1.7 million suspected cases in the Western hemisphere alone. There are currently no antivirals for treatment of CHIKV infection and novel anti-alphaviral compounds are badly needed. nsP1 is the alphavirus protein responsible for the methyltransferase and guanylyltransferase activities necessary for formation of the 5’ type 0 cap structure added to newly formed viral RNA. Formation of this cap depends on nsP1 binding GTP and transferring a methylated GMP to nascent viral RNA. We have developed a fluorescence polarization-based assay that monitors displacement of a fluorescently-labeled GTP analog in real time. Determining the relative affinities of 15 GTP analogs for nsP1 GTP revealed important structural aspects of GTP that will inform identification of inhibitors able to outcompete GTP for the nsP1 binding site. Validation of the assay for HTS was completed and a secondary orthogonal assay that measures guanylation activity was developed in order to evaluate hits from future drug screens. This platform provides an avenue for identification of potent nsP1 inhibitors, which would potentially provide compounds capable of treating disease caused by CHIKV infection.

## Introduction

Chikungunya virus (CHIKV) is an Old World alphavirus from the family *Togaviridae*, genus *Alphavirus*. CHIKV is transmitted by the bite of an infected female *Aedes* mosquito and causes debilitating disease symptoms including but not limited to fever, rash, and sever joint pain, which may persist in some cases for months or years post-infection [1,2]. CHIKV transmission has been documented since 1953 and was mainly found in low levels in Asia, Africa and the Indian subcontinent [3]. However, several factors including the recent habitat expansion of the mosquito vector have led to spread of the disease and an alarming transmission rate in the Western hemisphere [4,5]. To date there have been an estimated 1.7 million suspected cases of CHIKV infection reported in the Caribbean, Latin America and the United States combined and 191 CHIKV associated deaths [6]. As such, efforts to combat the spread of CHIKV have redoubled. Unfortunately, there are no existing commercially available therapeutics for the treatment of CHIKV infection, thus clinicians must rely solely on symptom remediation when treating infected individuals. There is an immediate need to identify and pursue promising anti-CHIKV drug targets in order to identity novel or existing compounds capable of treating this disease.

CHIKV has an 11.6 kb positive sense single-stranded RNA genome with a 5’ type 0 cap structure and a 3’ poly-A tail [7–9]. After entry into the cell, two thirds of the viral RNA 5’ end is translated into a large polyprotein that is later cleaved into four nonstructural proteins and the remaining viral RNA gives rise to the structural polyprotein. The nonstructural proteins (nsP1-nsP4) assemble into the replication complex, which is responsible for synthesis of new genomic and subgenomic RNAs (as reviewed in [10]). While the intricacies of alphavirus capping are still being uncovered, the general mechanism of RNA capping begins when nsP2 acts as a 5’ RNA triphosphatase and cleaves the terminal phosphate from nascent viral RNA, leaving a 5’ diphosphorylated RNA molecule [11]. Methyltransferase activity of the nsP1 protein then transfers a methyl group from S-adenosylmethionine to GTP. nsP1 forms a nsP1-me^7^GMP intermediate before finally transferring the covalently linked me-^7^GMP to the diphosphorylated viral RNA to form the mature type 0 RNA cap structure. This RNA cap is vital for RNA replication as it directs translation of the viral polyprotein and along with part of the 5’ UTR of the viral RNA, protects the viral genome from being degraded by host cell RNases, and engages the host cell’s immune response [12]. Thus, the function of proteins that comprise the viral RNA capping machinery have become a promising point of therapeutic intervention for treatment of alphavirus-induced diseases such as CHIKV infection (as reviewed in [13]). The alphavirus nsP1 protein in particular is an attractive drug target because the virus cannot replicate without its capping activities [14] and because blocking GTP binding of other viral capping enzymes has previously lead to the identification of compounds with antiviral activity [15–18].

nsP1 is a 535 amino acid protein that is proteolytically cleaved from the viral polyprotein during positive strand synthesis to yield the active protein form [19]. nsP1 contains a membrane anchor helix that attaches the protein to the cytosolic surface of endosomal membranes where the viral replication complex assembles and nsP1 serves to stabilize the nsP1-3 complex for genome replication. Studies of nsP1 function in Sindbis and Semliki Forest virus (SFV) first revealed its N7 methyltransferase activity [20–22]. The first step in the guanylyltransferase reaction, the formation of the nsP1-GMP intermediate, was also first described with Sindbis and SFV nsP1 [23] and the full alphavirus guanylyltransferase reaction including transfer of the methylated GMP from nsP1 to the 5’ end of viral RNA was demonstrated for the first time recently with Venezuelan equine encephalitis virus nsP1 [24]. While cellular methyltransferase and guanylyltransferase enzymes often contain canonical motifs (KDKE and KxDG, respectively), nsP1 does not contain either signature motif [25] and identification of its activities have been entirely empirical. The lack of recognizable motifs and mechanistic differences that exist between the processes of viral and cellular RNA cap formation suggest it may be possible to identify molecules that would selectively inhibit viral nsP1 while leaving the activity of host cell capping enzymes unaffected. Therefore, developing a platform to identify potent inhibitors of CHIKV nsP1 activity has the potential to identify promising anti-alphaviral compounds. However, a sensitive and effective high-throughput screening (HTS) system to identify inhibitors of alphavirus nsP1 function has yet to be described.

In this report we describe a robust fluorescence polarization (FP)-based HTS assay capable of monitoring the ability of small molecules to compete for the CHIKV nsP1 GTP binding site in real time. First, we developed an efficient expression and purification scheme for CHIKV nsP1 in order to obtain monomeric protein that was soluble, pure and of sufficient quantity for HTS. We next determined optimal assay conditions including pH, DMSO tolerance, ideal volume, and stability over time. We performed statistical analyses on assay results for measures of plate uniformity, signal variability, and reproducibility. The resulting analysis indicated that our assay is robust with a Z’ prime score of >0.9, a Minimum Significant Ratio of <3, and Limits of Agreement between 0.33 and 3.0. These results suggest that our assay would perform well in a HTS campaign aimed at screening small molecules against the CHIKV nsP1 protein. We tested a number of nucleotide analogs for their ability to displace GTP-bodipy from CHIKV nsP1 to begin identifying structural components of GTP that aid in protein binding. Additionally, we established a secondary orthogonal assay able to detect the degree to which small molecules inhibit nsP1 guanylation activity as evidenced by the formation of a covalent bond between nsP1 and a fluorescently-labeled GTP analog.

## Materials and Methods

### Expression and purification of CHIKV nsP1

A codon-optimized full length recombinant CHIKV nsP1 gene (La Reunion strain, Genebank: FR717337.1, aa 1–535) with an C terminal GSSS-6-histidine tag was synthesized (Genescript) and cloned into a T7 expression plasmid. The CHIKV nsP1 construct was transformed into Gro7 (Takara no. 3340) BL21 (DE3) *E*. coli competent cells (Novagen). Resulting transformations were transferred into 5 mL culture tubes of 2X YT media supplemented with ampicillin (50 μg/mL), and chloramphenicol (34 μg /mL). Starter cultures were grown overnight at 37°C and then used to inoculate 100 mL cultures of 2X YT media with the same antibiotic selection and trehalose (1%). These larger cultures were allowed to grow overnight at 22°C with shaking. The following day, cultures were transferred into 1L baffled flasks, containing 2X YT media (750 mL) with the same antibiotic selection, trehalose (1%), and L-Arabinose (500 μg/mL) to induce expression of the Gro7 chaperone. Cultures grew for 30 min at 22°C with shaking and were then induced with 400 μM IPTG. Cultures grew for an additional 4 hours at 22°C with shaking before being collected by centrifugation at 5000 rpm for 20 min, resuspended in 25 mL low imidazole buffer (LIB) (50 mM Tris pH 6.8, 400 mM NaCl, 20 mM imidazole, 5% glycerol and 1 mM TCEP HCl) supplemented with protease inhibitor cocktail (Roche), and stored at -80°C.

For purification, frozen bacterial pellets were thawed and added to 25 mL lysis buffer (50 mM Tris pH 6.8, 400 mM NaCl, 5% glycerol, 1% CHAPS, 1 M trehalose and 1 mM TCEP HCl) supplemented with protease inhibitor cocktail. Three 750 mL cultures were combined, disrupted 3 times on a 110S Microfluidizer (Microfluidics), clarified by centrifugation for 20 minutes at 17,000 rpm at 4°C, and particulates were removed with a 0.45 μM syringe filter. His-tagged CHIKV nsP1 was purified from clarified bacterial lysates with immobilized metal affinity chromatography using a HisTrap FF (GE) column on an AKTA Pure FPLC system (GE). Proteins were eluted with with a gradient of high imidazole buffer (50 mM Tris pH 6.8, 400 mM NaCl, 500 mM imidazole, 5% glycerol and 1 mM TCEP HCl). Protein was further purified using size exclusion chromatography on a HiLoad 16/600 Superdex 200 pg (GE) gel filtration column in gel filtration buffer (50 mM Tris pH 6.8, 400 mM NaCl, 20% glycerol and 1 mM TCEP HCl). Protein was concentrated using Vivaspin Turbo 15 (Sartorius) ultrafiltration spin columns, divided into single use aliquots, and stored at -80°C until use.

### FP assay optimization

All FP assays were done in black, low binding 384-well plates (Corning 3573). Protein was incubated Bodipy-**γ**-phosphate-labeled GTP analog (Thermo Fisher Scientific G22183) or Bodipy-**γ**-phosphate-labeled ATP analog (A-22184) and plates were scanned for total fluorescence and fluorescence polarization using a Victor X5 multilabel plate reader (Perkin Elmer) (Excitation: 488 nm / Emission: 535 nm).

#### pH sensitivity

In order to determine pH sensitivity of the assay, CHIKV nsP1 (final concentration 1.5 μM) was incubated with buffer (Either 50 mM HEPES pH 6.8, Tris pH 6.8, Tris pH 7.5, Tris pH 8, Tris pH 8.5 or CHES pH 9 and 0.01% NP-40), 10 nM GTP-Bodipy and 2 mM DTT for bound GTP-Bodipy reading. Free bodipy control wells contained buffer conditions matched to those stated above, 10 nM GTP-Bodipy and 2 mM DTT. Each condition and controls for each condition were plated in triplicate and plates were incubated in the dark at 25°C for 1 hour before reading. Signal window (difference between free vs. bound GTP-Bodipy FP readings) was calculated for each buffer condition.

#### DMSO tolerance

CHIKV nsP1 (final concentration 500 nM) was incubated in FP binding buffer (50 mM HEPES pH 6.8, 0.01% NP-40), 10 nM GTP-Bodipy and 2 mM DTT and indicated DMSO concentrations ranging from 10% to 0.01% for bound GTP-Bodipy reading. Free bodipy wells contained FP binding buffer, 10 nM GTP-Bodipy, 2 mM DTT and indicated DMSO concentrations ranging from 10% to 0.01%. Assays were plated in 50 μL final volume per well and plates were incubated in the dark at 25°C for 1 hour before reading. Each condition was plated in triplicate and signal windows (difference between free vs. bound GTP-Bodipy FP readings) were calculated for each DMSO concentration. Independent student t-tests were used to determine if there was a significant difference in signal window at each DMSO concentration compared to control wells containing no DMSO.

#### Assay volume

CHIKV nsP1 (final concentration 500 nM) was incubated in FP binding buffer, 10 nM GTP-Bodipy and 2 mM DTT. This mixture was plated at volumes ranging from 65 μL to 10 μL in triplicate. Plates were incubated in the dark at 25°C for 1 hour before reading. Signal windows were calculated for each volume.

#### Stability over time

CHIKV nsP1 (final concentration 1.5 μM) was incubated in FP binding buffer, 10 nM GTP-Bodipy and 2 mM DTT for bound GTP-Bodipy wells. Free bodipy wells consisted of FP binding buffer, 10 nM GTP-Bodipy and 2 mM DTT without protein. All wells were plated with a 50 μL final volume. Plates were incubated in the dark at 25°C and were scanned at time 0 and every 15 minutes thereafter for 120 minutes. Signal windows were determined and plotted over time.

### CHIKV nsP1 K_i_ and K_d_ determinations

All K_d_ and K_i_ assays were carried out in 50 μL volumes in 384-well black plates as previously described [26, 27]. For K_d_ determinations, reaction mixtures contained FP binding buffer, 10 nM GTP-Bodipy or 5 nM ATP-Bodipy (Thermo Fisher Scientific A-22184) (experimentally determined 80% fractional saturation of each fluorophore) and 2mM DTT. Purified CHIKV nsP1 was serially diluted in GF buffer in a 1.5 fold dilution series and 5 μL of protein was added to 45 μL of the reaction mixture to yield final concentrations of protein ranging from 3 μM to 1 nM. K_i_ determinations were carried out using purified CHIKV nsP1 and the ligand was GTP-Bodipy. GTP and RNA cap analogs for competition analysis were purchased from Jena Biosciences. Reactions consisted of FP binding buffer, 10 nM GTP-Bodipy, 2 mM DTT, and 500 nM protein. Small molecules were diluted in water in a 1.5 fold dilution series and 2.5 μL of small molecule was added to 47.5 μL of the reaction mixture. Plates were incubated in the dark at 25°C for 1 hour before reading for total fluorescence and FP with a Victor X5 multilabel plate reader (Perkin Elmer) for total fluorescence and FP. Results were analyzed with nonlinear regression using GraphPad Prism version 6 software and K_i_ values were calculated as described previously [28].

### Validation of assay for HTS

Determination of assay variability and plate uniformity was carried out and analyzed using the methods and equations described in the HTS Assay Validation Manual [29]. Briefly, in order to determine the extent of FP signal variability over multiple days and to establish that FP signal was uniform within the screening plates regardless of well position, we set up three plates on three different days using an interleaved plate format. All reagents were prepared fresh on each of the three days and three types of FP conditions were plated in each plate in alternating columns until the plate was filled. Maximum FP signal wells contained recombinant CHIKV nsP1 (500 nM final concentration), FP binding buffer, 10 nM GTP-Bodipy, 2 mM DTT and 0.2% DMSO. Midpoint signal wells also contained 6.8 μM GTP (the determined EC_50_ concentration of GTP) and minumum signal wells contained FP binding buffer, 10 nM GTP-Bodipy, 2 mM DTT and 0.2% DMSO only. Plates were incubated in the dark at 25°C for 1 hour before reading for total fluorescence and FP with a Victor X5 multilabel plate reader (Perkin Elmer). Outliers on each plate were counted and mean, standard deviation and critical value of the mean were also determined. For each midpoint signal well we calculated percent activity and percent inhibition. In addition, the mean and standard deviation were calculated for midpoint signal on each plate. Z’ factor scores were calculated for each plate used in HTS validation as well as for K_i_ determination plates. Reported Z’ factor score is the resulting mean from all plates. FP response on each plate for different days was plotted in a scatter plot with FP signal on the y axis and wells arranged by row, then column or by column, then row to look for edge effects and drift. In order to assure the assay yielded repeatable results over a range of potencies, 11 GTP cap analogs were chosen with potencies ranging from 133 μM to 402 nM. Each compound was tested twice in two independent runs and EC50s were determined as described above. Results were analyzed and assay repeatability was determined according to the method described by Iverson et al.[29].

### Guanylation assays

Guanylation assays were performed in 10 μL final voumes as described in Stahla-Beek et al. 2012 [15]. Briefly, recombinant CHIKV nsP1 (3 μM final volume) was incubated with 1 μM 8-[(6-Amino)hexyl]-amino-GTP—ATTO-680 (Jena Bioscience no.NU-830-690) in guanylation buffer containing 50 mm HEPES pH 6.8, 500 nM MgCl_2,_ 0.1% NP-40 for 1 hour at 37°C in the dark. Resulting reactions were resolved on 12% SDS-PAGE gels at 150V for 1 hour. Gels were scanned on the 700 channel of an Odyssey fluorescence scanner (Li-Cor) and then stained with Coomassie blue. Coomassie gels were imaged with a ChemiDoc MP Imaging System (Bio-Rad). Fluorescence was quantitated with Image Studio Software (Li-Cor) and ImageJ was used to determine protein quantitiy in each gel band for fluorescence signal normalization. Each reaction was conducted in triplicate.

## Results

### Expression and purification of enzymatically active CHIKV nsP1

**Fig 1:**
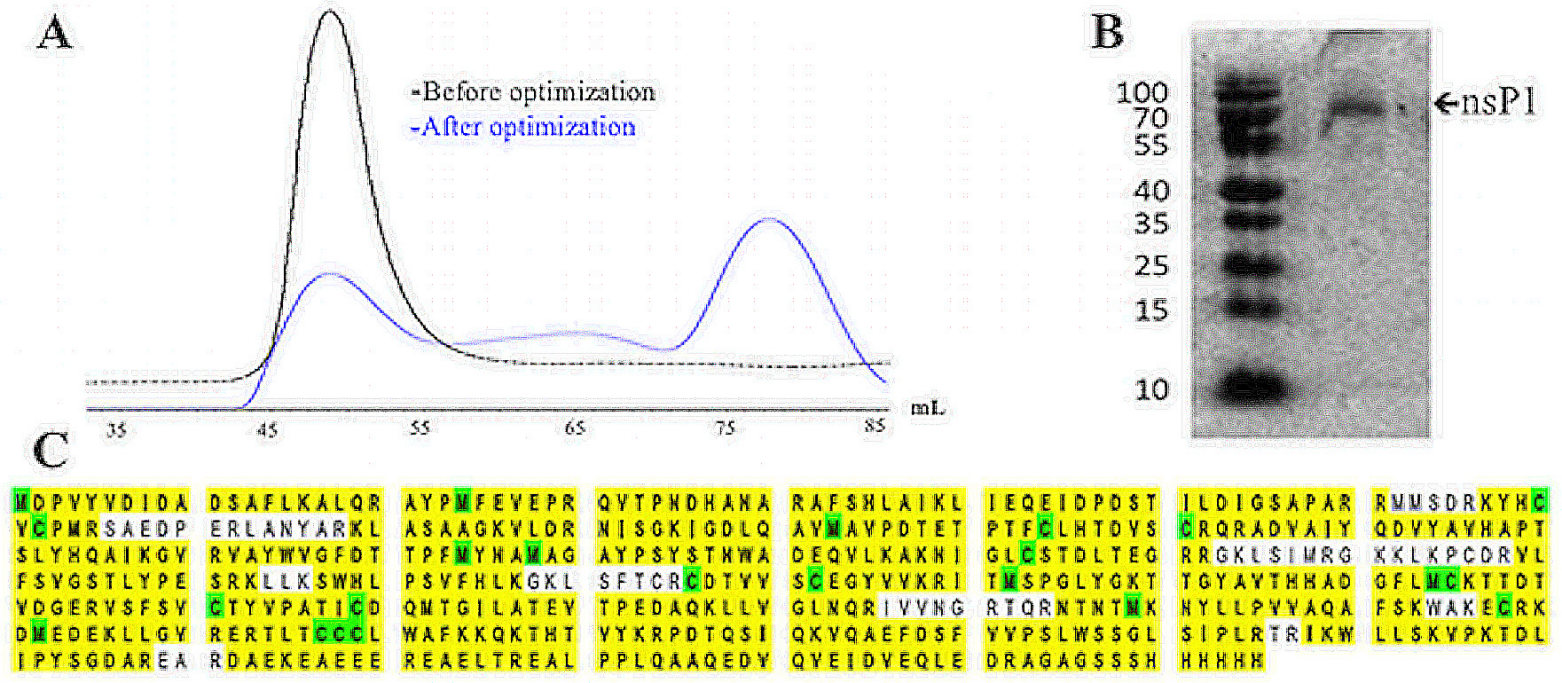
Validation of CHIKV nsP1 purity and identity. Recombinant CHIKV nsP1 was purified with 6X his-mediated affinity chromatography followed by size exclusion chromatography. (A) Early attempts to purify CHIKV nsP1 resulted in highly aggregated protein that eluted exculsively in the void volume (eluted around 55 mL) during gel filtration, while protein purified after optimization of expression and purification conditions eluted around 72 mL. (B) Eluates resulting from size exclusion chromatography were concentrated and analyzed with SDS-PAGE gel electrophoresis and Coomassie staining. (C) This band was cut out, digested with trypsin and analysed further with orbitrap mass spectrometry analysis.

In order to determine the optimal expression system for CHIKV nsP1, the GSSS 6-histidine tagged, codon optimized nsP1 construct was chemically synthesized and transformed into BL21 (DE3) pLysS bacterial cells. While CHIKV nsP1 expressed well in this system and was found exclusively in the soluble fraction, early attempts to purify the protein by gel filtration revealed the protein was still highly aggregated as indicated by protein eluting solely in the void volume during size exclusion. In order to increase proper protein folding and reduce aggregation, BL21 (DE3) cells were cotransformed with Gro7 chaperone (Clontech Laboratories). Further optimization of expression length and temperature resulted in higher yields of monomeric nsP1 after size exclusion chromatography and addition of chemical chaperone D-(+)-trehalose in bacterial cell lysis buffer and expression media further improved yields of monomeric protein (Fig 1A). Following the optimization of expression and purification protocols for CHIKV nsP1, large-scale production of the protein was conducted as indicated in the ‘materials and methods’ portion of this paper.

CHIKV nsP1 with a C-terminal 6X histidine tag was purified from bacterial lysates using a two-step purification method involving immobilized nickel affinity chromatography to first isolate His-tagged protein from cellular proteins followed by size exclusion chromatography. Resulting protein was concentrated to approximately 90 μM (~2 mL per 2 L bacterial culture). Concentrated protein samples were analyzed by SDS-PAGE, stained with Coomassie and judged to be >95% pure (Fig 1B). The resulting 61 kD band was cut from the gel, digested with trypsin, and analyzed by Orbitrap mass spectrometry (Velos) at the Colorado State University Proteomics and Metabolomics Facility. Mass spectrometry analysis revealed that the mass of the protein was 61088.0 Daltons and it contained the expected C-terminal 6X-histidine tag (Fig 1C). The protein was identified as CHIKV nsP1 with 89% coverage of the amino acid sequence.

**Fig 2:**
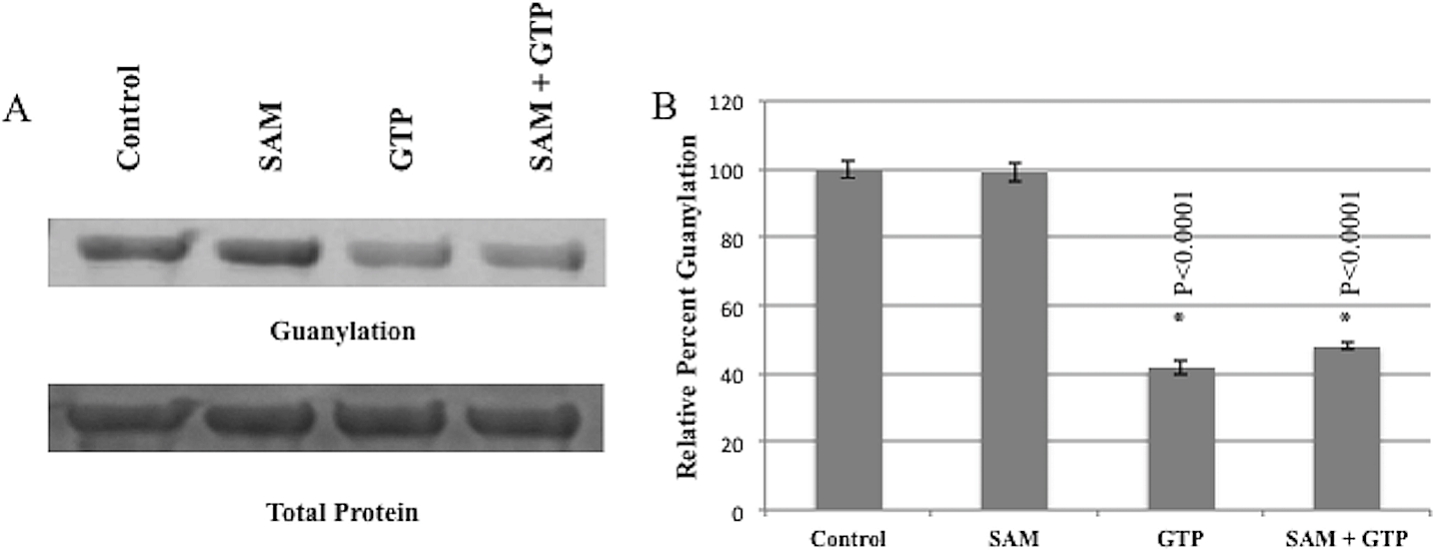
Purified CHIKV nsP1 is enzymatically active. Purified CHIKV nsP1 was incubated with GTP-680 and either 100 μM GTP, 100 μM SAM or 100 μM SAM and GTP for 1 hour. Reactions were resolved on SDS page gels and gels were scanned for fluorescence on an Odyssey Clx Infrared Imaging System before being stained with Commassie. (A) Guanylation was quantified and normalized to protein quantity using ImageJ and Image Studio software. Analysis of guanylation signal indicated a robust signal in Control and SAM only wells and depressed guanylation signals in GTP and GTP + SAM wells (gel shown is a representative gel). (B) Percent of control was calculated for Control, SAM, GTP and GTP + SAM wells. n = 3.

In order to determine if purified CHIKV nsP1 was enzymatically active, we tested its ability to perform the guanylation step of the guanylyltransferase reaction. Protein was incubated with 8-[(6-Amino)hexyl]-amino-GTP—ATTO-680 (GTP-680) and either GTP, S-adenosyl methionine (SAM) or both GTP and SAM for 1 hour. Reactions were resolved on an SDS-Page gel and read for fluorescence on an Odyssey CLx Infrared Imaging System to determine the extent of nsP1 guanylation. Purified CHIKV nsP1 was able to form a covalent bond between itself and GTP-680 (Fig 2) (S1 Supporting Information. Data for Fig 2). This guanylation activity was maintained in the presence of SAM alone, but addition of GTP significantly diminished guanylation activity as did the addition of both GTP and SAM. Taken together these data indicate that recombinant CHIKV nsP1 has guanylation activity, that guanylation activity is not SAM dependent in this system, and that nsP1 guanylation activity can be inhibited by the addition of unconjugated GTP.

### FP Assay Optimization

**Fig 3:**
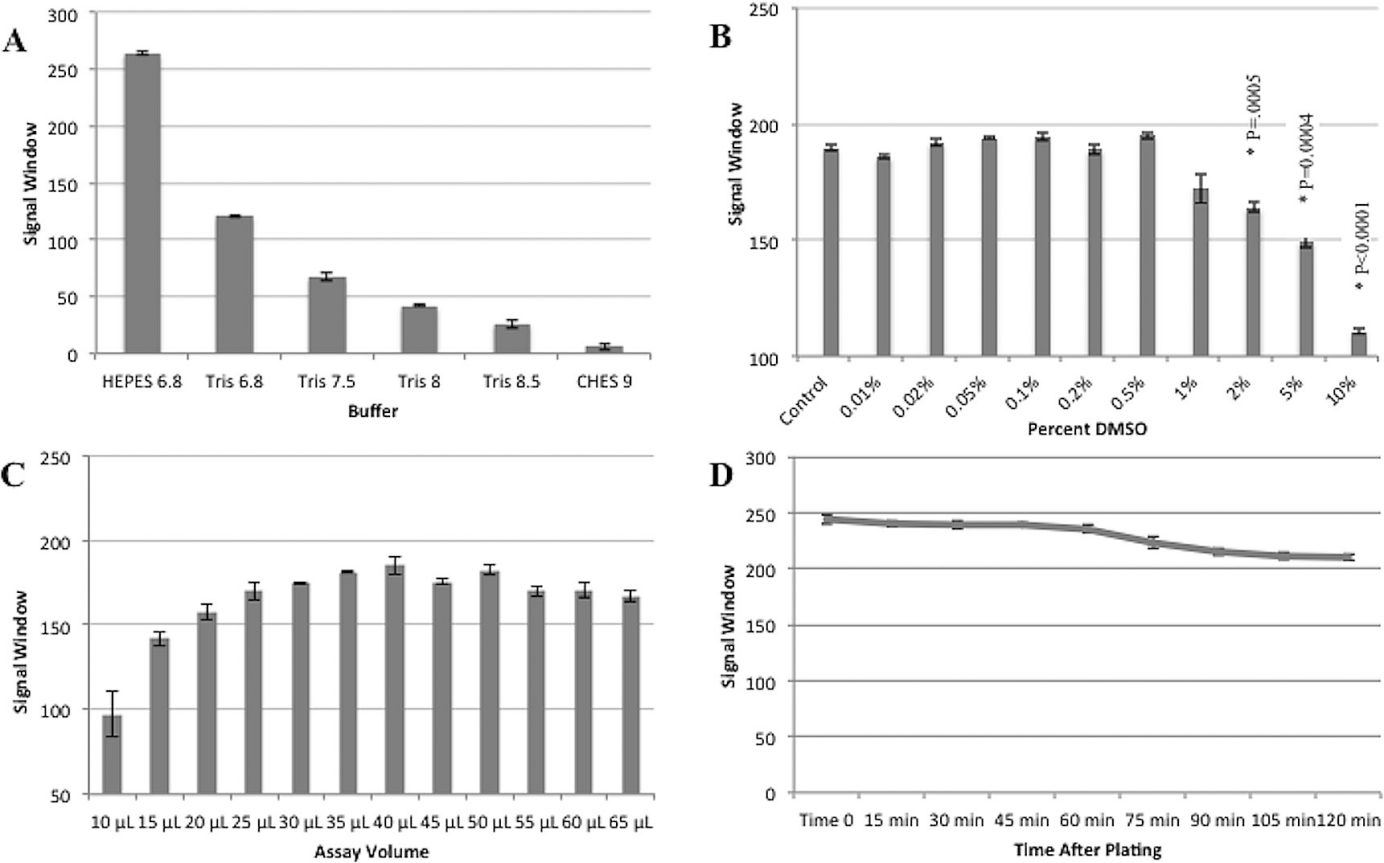
Optimization of FP assay for CHIKV nsP1. In order to determine the optimal pH for the FP assay, purified CHIKV nsP1 was incubated in the indicated buffer types with 10 nM GTP-Bodipy in black plates for 1 hour at 25°C in the dark. (A) Plates were then scanned for FP and signal windows were calculated for each buffer condition. (B) To determine the effect of DMSO concentration on FP assay signal window, purified CHIKV nsp1 was incubated with 10 nM GTP-Bodipy and various concentrations of DMSO ranging from 0.01%-10%. Plates were incubated for 1 hour at 25°C in the dark and then read for FP. (C) In order to determine the optimal assay volume, purified nsP1 was incubated with 10 nM GTP-Bodipy in FP binding buffer with 0.2% DMSO and plated in black plates in decreasing volumes from 65 μL to 10 μL. Plates were incubated for 1 hour at 25°C in the dark, scanned for FP and signal windows were calculated for each volume. (D) In order to determine the stability of the signal window over time, CHIKV nsP1 was incubated with 10 nM GTP-Bodipy at 25°C in the dark. Plates were scanned every 15 minutes for 120 minutes and signal windows were calculated at each time point. n = 3.

Failure to determine ideal assay parameters and assess assay stability during initial development stages could result in the identification of false hits, inability to detect active compounds or simply the lack of a consistent signal to noise ratio. Any of these potential issues could result in a significant loss of time and research funds which can little be afforded given the immediate need in the area of CHIKV drug discovery. In order to avoid these scenarios, assay conditions were optimized to maintain sufficient signal window and assay stability using GTP-bodipy concentrations previously optimized for FP assays [27] (S2 Supporting Information. Data for Fig 3.). Optimal pH and buffer conditions were identified by incubating purified nsP1 in various buffer conditions with 10 nM GTP-Bodipy for 1 hour. Signal window was calculated by determining the difference in FP signal between free GTP-Bodipy and GTP-Bodipy bound to nsP1. The FP signal window increased towards more acidic pHs and interestly, though both Tris and HEPES buffers were tested at pH 6.8, the signal window was dramatically larger when protein was incubated in HEPES buffer (Fig 3A). Further optimization of the FP assay was conducted with FP binding buffer containing HEPES buffer at pH 6.8 as this condition yielded the best signal window. DMSO tolerance was determined by incubating purified protein with 10 nM GTP-bodipy in binding buffer and increasing concentrations of DMSO from 0.01%-10%. Though the FP signal window began to decrease after 0.5% DMSO addition the difference did not become significant until 2%, so concentrations of DMSO of <1% would be useable for HTS with this assay (Fig 3B). Optimal assay volume was determined by adding purified nsP1 to FP binding buffer with 10 nM GTP-Bodipy. This mixture was plated in a range of volumes from 65 μL to 10 μL in triplicate and signal windows were calculated for each volume. FP signal window was relatively stable from 65 μL to 25 μL afterwhich the signal window steadily decreased and read variability increased (Fig 3C). A volume of 50 μL was chosen for future assays. Finally, assay stability over time was assessed by incubating purified CHIKV nsP1 with 10 nM GTP-Bodipy in FP binding buffer and measuring FP readings every 15 minutes for 90 minutes. The signal window remained relatively stable for the first 60 minutes of readings, but after 60 minutes the signal window began to steadily decrease (Fig 3D). As a result, all remaining assays were read after 60 minutes to allow sufficient time for small molecule equilibration and to maximize signal window.

### GTP and RNA cap analog competition analysis with CHIKV nsP1

In order to determine the degree of specificity with which nsP1 binds substrates, the K_d_ of GTP-Bodipy and ATP-Bodipy were determined by incubating increasing concentrations of purified nsP1 with either GTP-Bodipy or ATP-Bodipy for 1 hour and reading for fluorescence polarization. Fluorescence polarization was plotted against the log molar concentration of purified nsP1 and nonlinear regression analysis was used to calculate K_d_ values of the two fluorescently labeled nucleotide analogs. The affinity of CHIKV nsp1 for GTP-Bodipy was nearly 21 times greater than the affinity of CHIKV nsP1 for ATP-Bodipy with calculated K_d_ values of 100 nM and 2.1 μM, respectively (S5 Supporting Information. K_d_ and K_i_ calculations).

Following the K_d_ determination for GTP-Bodipy, the apparent K_i_ values for several GTP analogs and RNA cap analogs were established by incubating purified nsP1 with increasing concentrations of each analog and GTP-Bodipy for 1 hour, and then determining how each analog affected the fluorescence polarization signal. Log molar concentrations for each analog were plotted against fluorescence polarization values, and nonlinear regression analysis along with previously validated K_i_ conversion equations [28] were used to calculate apparent K_i_ values. These competition-based assays were used to describe structural aspects of GTP that are important for binding to the GTP-binding site of nsP1 (Table 1) (S5 Supporting Information. K_d_ and K_i_ calculations).

**Table 1:** GTP and RNA cap analog competition analysis with CHIKV nsP1.

GTP Analog	Average *Ki*	Standard Error	Fold Change
**XTP**	4.02E-07	3.20E-08	0.74
**ddGTP**	4.25E-07	4.68E-09	0.78
**GTP**	5.46E-07	2.19E-09	1
**me**^**7**^**-GTP**	1.55E-06	2.32E-07	2.84
**GpppA**	1.89E-06	3.81E-07	3.46
**ITP**	1.91E-06	2.11E-08	3.50
**ATP**	3.34E-06	2.78E-07	6.12
**dGTP**	4.21E-06	8.75E-08	7.71
**UTP**	4.46E-06	1.90E-07	8.17
**GDP**	4.89E-06	8.59E-07	8.96
**CTP**	6.94E-06	9.94E-07	12.71
**me**^**7**^**-GpppA**	7.00E-06	1.15E-06	12.82
**me**^**7**^**-GMP**	2.19E-05	4.42E-06	40.11
**GMP**	1.33E-04	5.08E-09	243.59
**Guanosine**	NA	NA	NA

First we assessed the ability of GTP to compete with GTP-Bodipy for the CHIKV nsP1 GTP binding site and found that GTP and GTP-Bodipy had similar affinities. This similarity in affinities validates the use of GTP-Bodipy as the substrate in our assay because small molecules able to outcompete GTP-Bodipy for the CHIKV nsP1 GTP binding site would effectively indicate the small molecule’s ability to compete for GTP in the binding site as well. We next evaluated any change in affinity resulting from modification to the base of the GTP analog. Replacing the carbonyl group extending from the C6 position in GTP with the amine group in ATP resulted in a six fold affinity reduction, while XTP, which possesses keto groups at both the C2 and C6 positions of the purine ring, had a slightly higher affinity compared to GTP. Removal of an amine group in the C2 position in ITP also resulted in a moderate decrease in affinity and replacement of a purine base with a pyrimidine base as in CTP and UTP resulted in a markedly decreased ability to outcompete GTP-Bodipy in the binding site.

We next examined the importance of the hydroxyl moities projecting from the sugar on GTP by comparing the affinities of dGTP, ddGTP and GTP. While dGTP had less affinity compared to the others, the difference in affinites between these analogs was relatively small with ddGTP binding slightly better than the others. In addition, we determined the impact of phosphate groups on binding by comparing the affinities of GTP, GDP, GMP and guanosine. There was a slight decrease in affinity when comparing GTP and GDP, while the decrease of GMP affinity was quite large and we were not able to calculate a K_i_ for guanosine. These data indicate that while the α-phosphate is somewhat important for binding, the β- and γ-phosphates are critical for maintaining the affinity of GTP for the binding site. Finally, as the function of nsP1 is to transfer me^7^GMP to the diphosphorylated 5’ end of viral RNA we investigated the ability of me^7^GTP as well as me^7^GMP to compete with GTP-Bodipy for the nsP1 binding site. Our results indicated that while me^7^GTP was able to compete for the GTP binding site, me^7^GMP was a very poor competitor. When we determined the K_i_s of cap analogs GpppA and me^7^GpppA we found that GpppA was a better competitor than me^7^GpppA. These results highlight the base specificity of CHIKV nsP1 as well as the importance of phosphate groups for GTP binding and may indicate that hydroxyl groups extending from the sugar moiety contribute little to binding. Insights gained from investigating different affinites of GTP analogs for the nsP1 GTP binding site may provide guidance for identifying ideal characteristics of potential nsP1 inhibitors.

**Fig 4:**
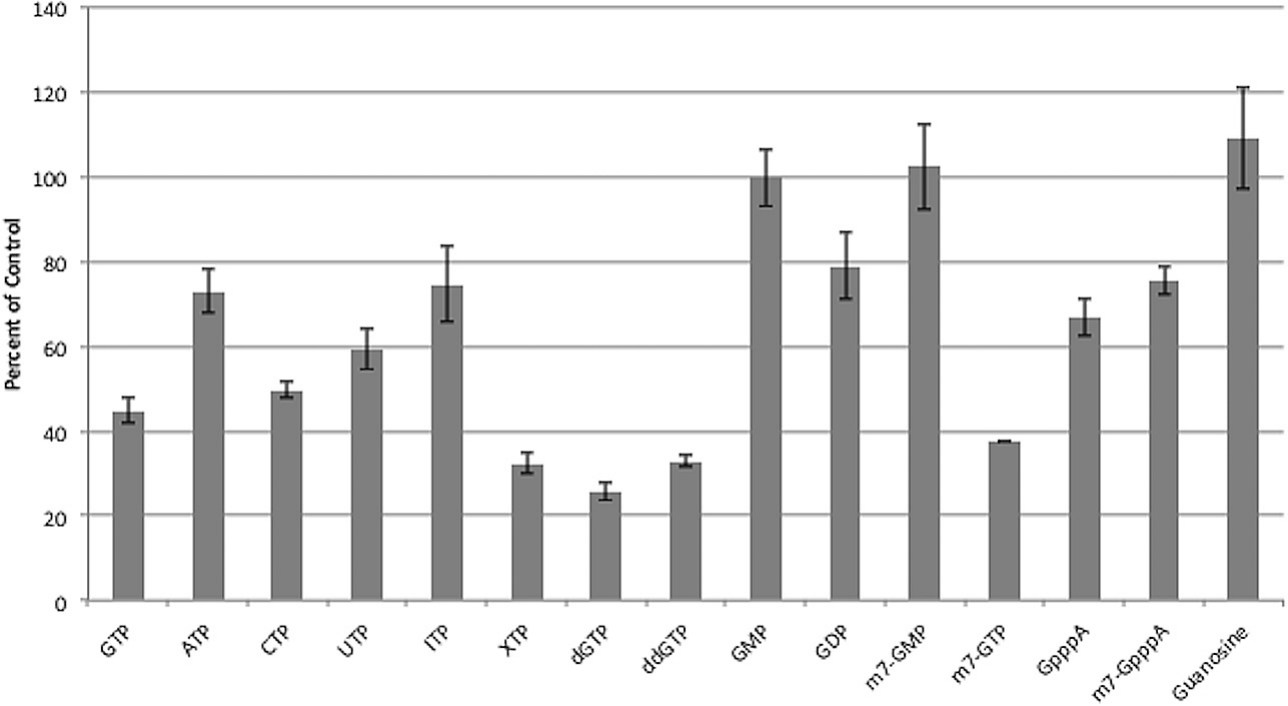
Inhibition of guanylation by GTP analogs. nsP1 was incubated with GTP-680 and 100 μM of each GTP analog for 1 hour. Reactions were resolved on SDS-PAGE gels, scanned for fluorescence on an Odyssey Clx infared imager, and then stained with Coomassie. Guanylation was quantified and normalized to protein amount using ImageJ and Image Studio software. Percent of control (containing no GTP analog) was calculated for each inhibitor. n = 3.

In addition to determining the ability of GTP analogs to outcompete GTP-bodipy, we also conducted an orthogonal assay which measures the ability of the CHIKV nsP1 protein to form a covalent bond with GTP-680 as a measure of guanylation activity. GTP analogs were evaluated for their ability to inhibit guanylation, which would be crucial for inhibitors of the enzyme. In general, there was good agreement between our FP assay results and our guanylation assay results (Fig 4) (S3 Supporting Information. Data for Fig 4.) with XTP, GTP, dGTP and ddGTP showing the greatest ability to in inhibit nsP1 guanylation activity and GMP, me^7^GMP and guanosine diplaying the least ability to inhibit guanylation. This secondary assay provides a useful tool for validating any hits from future drug screens with CHIKV nsP1.

### Validation of assay for HTS

**Fig 5:**
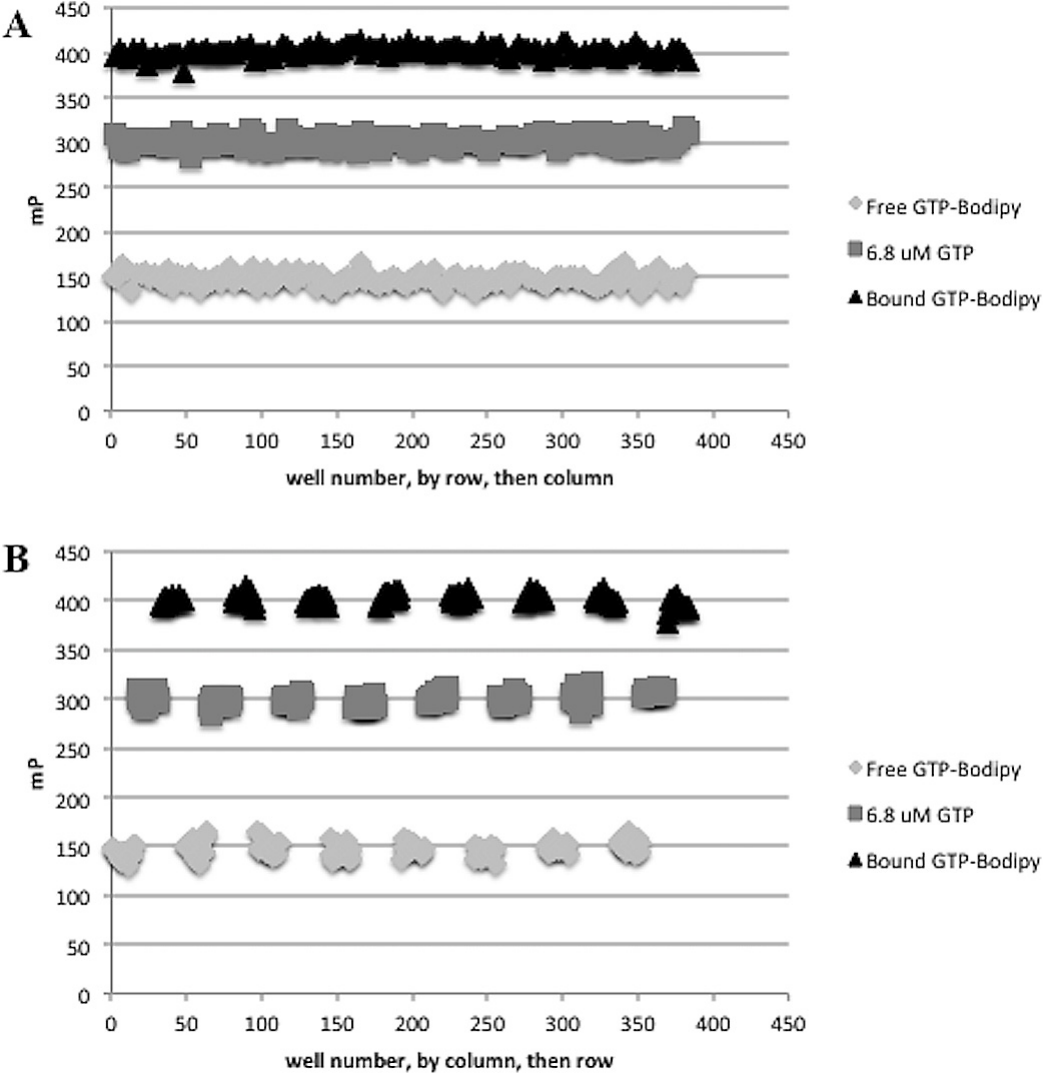
Drift and Edge Effects. In order to determine if there were any drift or edge effects, we used an interleaved plate format and plated three types of FP signals in alternating columns. Minimum signal wells contained 10 nM GTP-Bodipy in FP binding buffer only, midpoint signal wells contained CHIKV nsP1, GTP-Bodipy in FP binding buffer, and 6.8 μM GTP and maximum signal wells contained CHIKV nsP1 and GTP-Bodipy in binding buffer. Plates were incubated for 1 hour at 25°C in the dark and scanned for FP. Values were plotted on a scatter plot with FP signal on the y-axis and (A) well by row, then column on the x-axis and also with (B) well by column, then row on the x-axis. n = 3.

The FP assay was optimized for HTS using the method described in the HTS Assay Validation Guidance Manual [29]. Uniformity of signal within the screening plate was assessed over three days at differenct times of day using Min, Mid and Max FP signals plated in an interleaved format on a 384 well black plates. Plates were incubated for 1 hour before being read for total fluorescence and fluorescence polarization. Plates were evaluated for previously described acceptance critera [29]. Each plate used for the uniformity study contained less than 2% outliers. The CVs of each signal type were less than 20% using calculations for running test compounds in duplicate and standard deviations for percent activity of the midpoint signal (containing an EC_50_ concentration of unconjugated GTP) were less than 20 on all plates. Uniformity studies indicated no signal drift or edge effects (Fig 5) (S4 Supporting Information. Data for Fig 5).

We next determined how consistent the assay was at calculating EC_50_ values in order to verify that the results of our assay could be reproducable for small molecules with a range of potencies. As there are no commercially available GTP-competitive inhibitors for nsP1, we used 11 commercially available GTP analogs with a range of potencies and recorded EC_50_ curves for each analog in two independent runs plated in triplicate. We used the statistical analysis and acceptance critieria previously described to assess the consistancy of the assay [29]. A minimum significant ratio of 1.7 was calculated using EC_50_ values from the two runs indicating that within-run variability was within acceptable limits (< 3) and the limits of agreement were between 0.7 and 2.2 indicating acceptable between-run variability (between 0.33 and 3). Z’ factor scores were >0.9 on all plates used for both plate uniformity and potency studies. These data suggest this assay is sufficiently robust and would adapt well to a HTS format.

## Discussion

Given the lack of effective therapeutics for treatment of Chikungunya virus infection, developing methods capable of identifying compounds with anti-alphaviral activity is paramount. Targeting the CHIKV nsP1 GTP binding site is an excellent option for identifying compounds with anti-alphaviral activity because nsP1 has to bind GTP in order to cap newly formed viral RNAs and disrupting this activity prevents viral replication. We have described a robust and sensitive method for the identification of CHIKV nsP1 GTP competitive inhibitors and optimized this assay to perform in a HTS campaign with the goal of identifying compounds with anti-alphaviral drug activity. By determining the most favorable conditions for assay parameters such as pH, DMSO tolerance, assay stability and volume we have optimized the signal window of the assay. K_i_ determinations for a set of 15 GTP analogs allowed for formation of a preliminary strucutre activity relationship between GTP analogs and the nsP1 GTP binding site. Further, the ability of XTP and ddGTP to outcompete GTP for the nsP1 binding site in both the primary screening assay and the orthogonal assay indicates that identifying nsP1 modulators that bind to the protein with greater affinity than GTP is possible. Finally, the optimized assay was statistically validated for use in a HTS campaign and establishment of a secondary orthogonal nsP1 guanylation assay will allow for the followup of hits from drug screening. Identification of potent nsP1 inhibitors would provide badly needed compounds for the anti-alphaviral drug discovery pipline and potentially provide novel therapeutics for treatment of Chikungunya virus infections in the future.

## Supporting Information

S1 Supporting Information Data for Fig 2. (XLSX)

S2 Supporting Information Data for Fig 3. (XLSX)

S3 Supporting Information Data for Fig 4. (XLSX)

S4 Supporting Information Data for Fig 5. (XLSX)

S5 Supporting Information K_d_ and K_i_ calculations. (PZFX)
